# The ipsilateral motor cortex does not contribute to long-latency stretch reflex amplitude at the wrist

**DOI:** 10.1002/brb3.189

**Published:** 2013-11-28

**Authors:** Jonathan Fox, Jonathan Shemmell

**Affiliations:** School of Physical Education and Brain Health Research Centre, University of OtagoDunedin, New Zealand

**Keywords:** Flexor, posture, pyramidal tracts, stroke, upper extremity

## Abstract

**Background:**

A capacity for modulating the amplitude of the long-latency stretch reflex (LLSR) allows us to successfully interact with a physical world with a wide range of different mechanical properties. It has recently been demonstrated that stretch reflex modulation is impaired in both arms following monohemispheric stroke, suggesting that reflex regulation may involve structures on both sides of the motor system.

**Methods:**

We examined the involvement of both primary motor cortices in healthy reflex regulation by eliciting stretch reflexes during periods of suppression of the motor cortices contra-and ipsilateral to the extensor carpi radialis in the nondominant arm.

**Results:**

LLSRs were significantly attenuated during suppression of the contralateral, but not ipsilateral, motor cortex. Modulation of the LLSR was not affected by suppression of either primary motor cortex.

**Conclusion:**

Our results confirm the involvement of the contralateral motor cortex in the transmission of the LLSR, but suggest that the ipsilateral motor cortex plays no role in reflex transmission and that neither motor cortex is involved in stability-dependent modulation of the LLSR. The implications of these results for reflex impairments following stroke are discussed.

## Introduction

When operating in the physical world, our central nervous system must continually modify the stability of our body and limbs to compensate for instabilities in the environment. The requirement for lower limb stability is obvious when we try to walk on a slippery surface like ice, and the requirement for upper limb stability becomes evident during precision tasks such as writing, painting, or performing surgery. In all these tasks, it is important that any disturbance of limb posture be rapidly countered to avoid undesirable results. As humans, the most rapid neurophysiological mechanism we have available to regulate limb posture is the stretch reflex (Pearce 1997). The stretch reflex consists of several excitatory components (Hammond 1955) and has been attributed to the combined actions of multiple neural circuits. For example, in human forearm muscles the first component of the stretch reflex begins ∼20 msec after a muscle begins to elongate; this is termed the short-latency stretch response (SLSR) and is the most rapid component of the stretch reflex. Following this is a second response which occurs around 50 msec after the onset of muscle lengthening; this is termed the long-latency stretch reflex (LLSR; Hammond 1955). Given the rapidity of their action, these reflexive muscle responses represent our fastest defense against unexpected perturbations of limb or body position.

There is now a substantial body of evidence demonstrating that the sensitivity of the stretch reflex, particularly the LLSR, can be modified in response to changes in the amount of stability offered by the environment. Specifically, the amplitude of the LLSR is greater when individuals interact with compliant (less stable) environments than with stiff (more stable) environments (Doemges and Rack 1992; Perreault et al. 2008; Shemmell et al. 2009). Increasing the sensitivity of the LLSR in unstable circumstances enables the nervous system to respond to perturbations of posture or movement much faster than would be possible through the execution of voluntary corrective actions. Our understanding of which neural circuits are involved in regulating stretch reflex sensitivity, however, remains incomplete.

The neural pathway which contributes to the SLSR is a monosynaptic circuit consisting of Ia-afferent fibers, originating as stretch receptors in the intrafusal muscle fibers, and terminating in α-motoneurons which project back to innervate extrafusal fibers of the same muscle. The neural origin of the LLSR has not been definitively described, although there is convincing evidence to support the idea that the LLSR is initiated by the same muscle receptors as the SLSR, but traverses a longer neural pathway involving the motor cortex contralateral to the muscle of interest (Matthews 1991). The ascending branch of this pathway is likely to include afferent projections from the stretched muscle to the thalamus and/or area 3a within the primary sensory cortex, both shown to project directly to the primary motor cortex (Asanuma et al. 1979; Huerta and Pons 1990). Early evidence supporting the involvement of the primary motor cortex was obtained by observation in Rhesus monkeys of an increase in excitability of decussating corticospinal neurons originating in the primary motor cortex in response to perturbations of the wrist that stretched forearm flexor muscles (Cheney and Fetz 1984). It appears certain, therefore, that the primary motor cortex contralateral to a stretched muscle is involved in the transmission of the LLSR. More recent studies have also implicated the contralateral primary motor cortex in gain regulation for the LLSR, showing that transient suppression of motor cortex activity reduces the change in reflex amplitude observed between stable and unstable conditions (Kimura et al. 2006; Shemmell et al. 2009).

The results of a recent study examining reflex modulation following stroke, however, suggest that both cerebral hemispheres may have a role to play in the generation and gain regulation of the LLSR (Trumbower et al. 2013). Trumbower and colleagues (2013) have demonstrated that the expected changes in LLSR sensitivity with changes in environmental stability are not evident in either arm of individuals in the chronic phase of recovery after a monohemispheric stroke. These results suggest that reflex control in the “non-paretic” arm has been reduced through damage to the ipsilateral motor system. Although the majority of pyramidal tract neurons cross the midline at the cervicomedullary junction to innervate contralateral motoneurons (Landgren et al. 1962), noncrossing pyramidal tract neurons also exist in many mammals including rats, cats, monkeys, and humans (Armand and Kuypers 1980). In nonhuman primates, 8–13% of corticospinal fibers descending from the primary motor cortex do not cross the midline, instead synapse ipsilaterally with α-motoneurons or interneurons (Lacroix et al. 2004). Despite representing a small proportion of corticospinal tract fibers, stimulation of noncrossing axons is sufficient to excite α-motoneurons ipsilateral to the cerebral hemisphere from which they originate (Bernhard and Bohm 1954). Anatomical evidence also suggests that these noncrossing fibers influence the activity of motoneurons projecting to both proximal and distal muscles (Bernhard and Bohm 1954). In primates, noncrossing axons terminate on motoneurons and interneurons associated with the control of both proximal and distal muscles (Liu and Chambers 1964; Kuypers and Martin 1982; Lacroix et al. 2004). Despite the existence of noncrossing pyramidal tract neurons and extensive connections between homologous areas of the motor cortices (Lacroix et al. 2004) and spinal cord (De Lacoste et al. 1985), the potential for the primary motor cortex ipsilateral to a stretched muscle to play a role in regulating the LLSR has not been explored.

### Aims and hypotheses

The purpose of this study was to determine the role of each motor cortex (left and right) in the regulation of the LLSR recorded in the left wrist extensor muscle (extensor carpi radialis [ECR]). We formulated five specific hypotheses based on the results of previous studies: (1) that the amplitude of the LLSR elicited during interactions with a compliant (unstable) manipulandum would be larger than those elicited during interactions with a stiff (stable) manipulandum, (2) that inhibiting the contralateral (right) primary motor cortex would reduce the amplitude of the LLSR, (3) that inhibiting the ipsilateral (left) primary motor cortex would reduce the amplitude of the LLSR, (4) that inhibiting the contralateral primary motor cortex would reduce modulation of the LLSR between stiff and compliant mechanical environments, and (5) that inhibiting the ipsilateral primary motor cortex would reduce modulation of the LLSR between stiff and compliant mechanical environments. The findings of this study improve our understanding of the neural pathways mediating the LLSR and may inform the development of treatments following stroke.

## Methods

### Participants

Nine right-handed adults (four female, five male) aged 18–25 with no history of neurological impairment participated in the experiment. All participants were screened to ensure that they did not have any contraindications to transcranial magnetic stimulation (TMS) and all were determined to be right handed by scoring >40 on the Edinburgh Handedness Inventory (Oldfield 1971). Prior to their involvement in the experiment, each participant was informed about the techniques to be employed during the experiment verbally and in writing, before signing a consent form. All informed consent procedures were approved by the University of Otago Human Ethics Committee and were consistent with the Declaration of Helsinki.

### Apparatus

**Figure 1:**
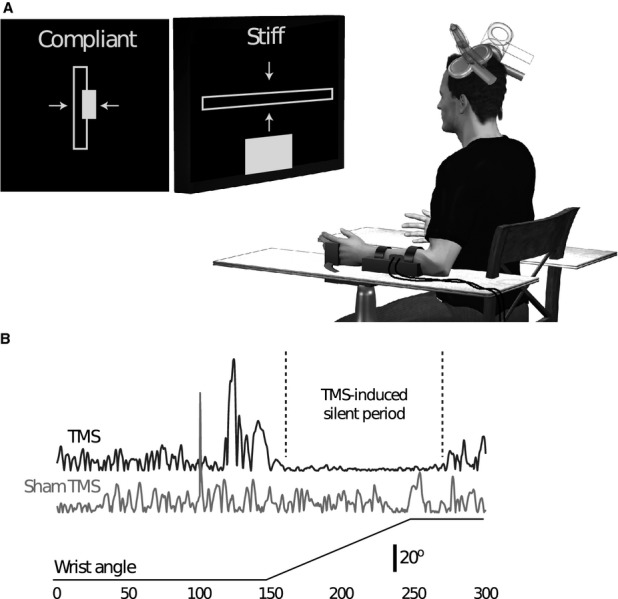
(A) Participantsx' forearms were placed in rests with their hand in a contoured holder connected to the servomotor. An LCD screen provided visual feedback of wrist torque and displacement. TMS was applied in real (flat on scalp) and sham (edge of coil on scalp) forms to each motor cortex at the optimal location for eliciting responses in the ECR of the left arm. (B) Responses of the ECR muscle to real and sham supramotor threshold TMS of the contralateral motor cortex are depicted. Real TMS is followed by an initial excitatory response, followed by a period of EMG silence termed the silent period. Sham stimulation applied at the same location and intensity elicits neither excitatory nor inhibitory responses from the same muscle. The bottom trace illustrates the timing and displacement of wrist flexion perturbations applied to generate stretch reflexes in the ECR muscle. TMS, transcranial magnetic stimulation; ECR, extensor carpi radialis; EMG, electromyograph.

Participants were seated comfortably facing a visual display monitor with their nondominant (left) forearm placed in a custom orthopedic restraint and secured with Velcro straps (Fig. 1A). Their nondominant forearm was held in a neutral position between maximum pronation and supination with the interior elbow angle at 90°. Wrist perturbations were applied by a custom-designed lever system attached to a servomotor, the rotational axis of which was positioned directly below the flexion/extension axis of wrist rotation. Custom computer software was used to control the characteristics of each perturbation (timing, duration, and amplitude) and the timing of each TMS pulse (Fig. 1B). The same software provided visual feedback to indicate the nature of the task (current and target forces/positions) and triggered auditory tones at quasirandom intervals during each trial designed to mask the sound of TMS discharge. The servomotor was instrumented with a potentiometer to provide position information and was configured during the appropriate portions of the study as either a stiff velocity and position servo (8.46 Nm resistance to movement) or a compliant load easily moved by the subject (0.53 Nm resistance to movement). These different mechanical environments were implemented using an admittance control algorithm implemented in Visual Basic. Perturbations were identically matched in each mechanical environment.

Participants were fitted with a breathable swimming cap and disposable electrodes (Ambu, Glen Burnie, MD) placed over the belly of the ECR longus muscle of each forearm to record muscle electrical activity. Electromyographic (EMG) signals were amplified and high-(1000 Hz) and low-pass (0.3 Hz) filtered online (Grass Technologies, Astro-Med, Inc., West Warwick, RI) before being converted into digital signals through a Powerlab™ data collection unit (ADInstruments, New South Wales, Australia), and monitored and stored on a computer. Transcranial magnetic stimuli (TMS) and sham TMS were applied in each trial with a Magstim 200^2^ stimulator (Magstim™, Whitland, Wales; see 1977 section below).

### Experimental task

Participants' capacity for torque generation about the wrist was measured by recording the largest torque produced in three successive maximal voluntary contractions (MVCs) of the wrist extensor muscles. In all subsequent trials, the target endpoint force level was set to 5 ± 1% MVC. Participants were provided with a visual display of wrist extension torque along with the target torque range (see Fig. 1A). Wrist extension perturbations and magnetic stimuli were delivered when the target torque had been maintained for 1 sec.

In this experiment, we evaluated the role of the contralateral and ipsilateral primary motor cortex in regulating the amplitude of the long-latency reflex in the ECR longus muscle to cope with changes in environmental stability. To do this we elicited LLSRs during a period of cortical suppression induced by TMS (Kimura et al. 2006; Shemmell et al. 2009). Reflexes were elicited in each participant with perturbations of the left (nondominant) wrist that were 45° in amplitude and occurred with a velocity of 450°/sec. The duration of each perturbation was therefore 100 msec, sufficient to elicit consistent long-latency responses in other upper limb muscles (Lewis et al. 2005). Twenty perturbations were applied in each of two task conditions: a condition in which subjects interacted with a stiff mechanical environment (stiff) and received an instruction of “Do not intervene” with the perturbation, and a mechanical environment with reduced stiffness (compliant) with the same instruction. In addition, TMS and sham TMS were applied over the motor cortical representations of the ECR muscle in both the contralateral (contra) and ipsilateral (Ipsi) cerebral hemispheres. In each trial, TMS (or sham TMS) was applied 50 msec before the wrist perturbation. The order of task conditions was randomized for each participant. During each block of 20 perturbations participants were provided with visual feedback of wrist torque, along with the target torque level (equivalent to 5 ± 1% MVC). The instantaneous wrist torque was displayed either as a horizontal (stiff) or vertical (compliant) bar on the screen, whereas the target force level was represented as a rectangle in the corresponding orientation that changed color when the target was achieved (Fig. 1A). Within each block, the mechanical environment and TMS type (sham or real) were randomized. The cortical hemisphere stimulated could not be randomized and was therefore constant within each block of trials.

Prior to each trial, participants were visually prompted as to whether the upcoming trial would involve a stiff or compliant environment. In the stiff condition, participants were required to apply and hold a target wrist flexion torque (5 ± 1% MVC) against the servomotor, in the compliant condition they were to hold their hands in a target zone of 0 ± 1° (i.e., a neutral flexion/extension position). After the target position or torque was held for 1 sec the computer software initiated a trial. To become familiar with the environment, participants were required to extend and flex their wrist before holding the target. Baseline levels of ECR muscle activity were matched in each environmental condition. In the test trials, perturbations were applied 50 msec after TMS or sham TMS in both the stiff and compliant haptic environments. In total there were eight possible combinations of task environment (stiff/compliant) and TMS (left TMS/right TMS/left sham/right sham). These were presented in pseudorandom order, each condition being tested before any condition was repeated. With TMS applied to one motor cortex, eight blocks of 20 trials (160 trials total) were completed with rest periods of at least 2 min between blocks to avoid muscle fatigue. Trials were separated by random intervals ranging from 3 to 8 sec. The order in which TMS was applied to each motor cortex was counterbalanced across participants.

### Cortical stimulation

TMS was applied to the primary motor cortex to induce cortical suppression during the period within which afferent information elicited by the muscle stretch would be traversing the cortex (Fig. 1B). TMS was administered with a MagStim 200^2^ (Magstim Company Ltd., Whitland, UK) via a figure-of-eight coil (coil diameter 70 mm). The coil was positioned over the subject's head with the handle pointing posteriorly and oriented ∼45° from the midsagittal line. The optimal site for stimulation over each cortical hemisphere was located by moving the coil in discrete steps across the scalp until the site eliciting the largest responses in the contralateral ECR muscle was located. The optimal stimulation site was marked on a lycra cap on the participants' head, and coil position was visually monitored by the operator during each experiment. The stimulation intensity was determined as the intensity at which a 150 msec period of EMG silence (as measured from the stimulus trigger) in the tonically active ECR (5% of MVC) was observed following the motor-evoked potential in 10 consecutive stimuli. The LLSR was timed to occur within the latter portion of the induced silent period (100–150 msec after TMS trigger) to evaluate cortical effects on the stretch response. This technique has previously been shown to reduce task-specific stretch reflex modulation without eliminating the reflex response, suggesting that it affects cortical neurons involved in regulating reflex sensitivity without disrupting the primary reflex pathway (Kimura et al. 2006; Shemmell et al. 2009). The existence of separable spinal-and cortical-level inhibitory effects within the TMS-induced silent period is informed by evidence that H-reflexes elicited within the silent period recover to baseline levels before the end of the silent period (Fuhr et al. 1991) and that stimulation of descending motor pathways at the level of the cervicomedullary junction induces a silent period of ∼50 msec, significantly shorter than that induced by TMS (Inghilleri et al. 1993). The application of TMS results in an auditory “click” that may have influenced the subjects' reaction time when instructed to resist an imposed perturbation. We controlled for this possibility by masking the sound of the TMS click with recordings of the same sound played at random intervals during each trial. We also included a sham stimulation condition in which the TMS coil was positioned on the scalp perpendicular to its most effective orientation (Fig. 1A). Although a magnetic field is still produced at the scalp in this orientation, its strength is far smaller than at the center of the coil and sham TMS applied in this manner failed to induce any excitatory or suppressive responses in the ongoing EMG trace from the ECR (Fig. 1B). This was true for all participants. Subjects were not aware of whether TMS would be applied in any given trial.

### Data processing and analysis

EMG recordings were rectified and averaged across all 20 trials in each experimental condition before further processing. All EMGs are expressed in mV of electrical activity recorded at the skin. Background EMG was quantified as the mean of the EMG within the period of 50–70 msec before perturbation onset, the period immediately before the application of TMS. The onset latencies of the short-and long-latency responses were determined visually within appropriate time windows (SLSR: 10–40 msec after perturbation onset, LLSR: 40–80 msec after perturbation onset). The onset latency of each response was determined from data obtained in sham TMS conditions and the same onset latencies assumed for trials with TMS. For each perturbation, long-latency response amplitudes were quantified as the mean of the rectified EMG signal over a 20 msec time window after response onset. For sham TMS trials, reflex amplitudes were quantified relative to the background EMG before perturbation onset. For TMS trials, reflexes were quantified relative to the mean EMG measured during the silent period of the TMS-only trials, corresponding to the time period used for reflex calculations. Levels of background activity were matched in each experiment, and trials were eliminated off-line if the background muscle activity exceeded the mean of 20 trials ± 1.5 SD (∼5% of trials). Paired *t-*tests were used to compare background EMG levels and reflex amplitudes between the experimental conditions specified in each hypothesis independently. Differences at an overall α level <0.05 were considered significant. Results are reported as mean ± SD.

## Results

**Figure 2:**
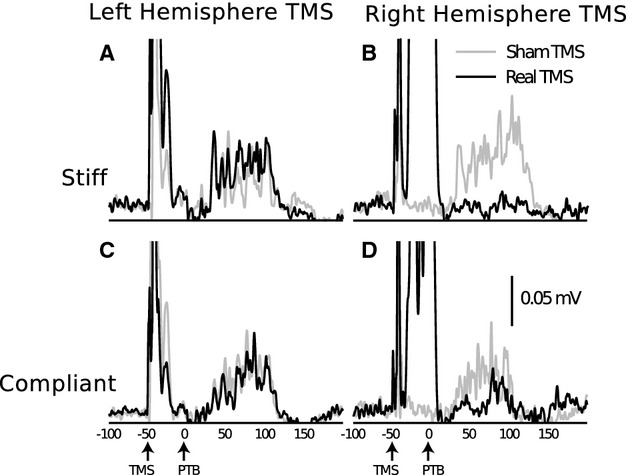
Responses of the ECR muscle in one individual to wrist flexion perturbations with and without preceding cortical stimulation. The traces shown are averaged across 20 trials in each condition. While real TMS applied to the left (ipsilateral) primary motor cortex has no effect on the amplitude of the LLSR in either the stiff (A) or compliant (C) mechanical environment, TMS applied to the right (contralateral) motor cortex substantially reduced the amplitude of the reflex response in both the stiff (B) and compliant (D) environments. The onset of TMS applications and wrist perturbations (PTB) is shown by arrows under the timescale. ECR, extensor carpi radialis; TMS, transcranial magnetic stimulation; LLSR, long-latency stretch reflex.

The application of TMS at supramotor threshold intensities reliably induces an initial excitatory response followed by a period of silence in the recorded muscle activity lasting up to 250 msec (Fuhr et al. 1991; Valls-Solé et al. 1992). We established a stimulation intensity for each participant that reliably achieved silent periods following stimulation of greater than 100 msec. Sham TMS was applied using the same TMS intensity so that the auditory effect of stimulation remained consistent across experimental conditions. Examples of responses to TMS and sham TMS are presented in Figure 1B. It is clear from Figure 1B that sham TMS did not elicit the same excitatory or inhibitory response in the target muscle as real TMS. Although the data shown are taken from one participant, the same pattern of EMG response to TMS and sham stimuli was observed for every participant. Real or sham TMS was followed in each trial 50 msec later by a wrist flexion perturbation that elicited a stretch reflex. Examples of the resultant EMG responses are shown in Figure 2 for a single participant. Changes in the amplitude of the elicited reflexes across the eight experimental conditions (two mechanical environments × two TMS positions × two TMS conditions) are addressed below according to hypothesis.

Hypothesis 1: that the amplitude of the LLSR elicited during interactions with a compliant manipulandum would be larger than those elicited during interactions with a stiff manipulandum.

When wrist perturbations were applied following sham stimulation the amplitude of the resulting LLSR was significantly greater when participants were interacting with a compliant manipulandum (0.1 ± 0.09 mV) than when the manipulandum was stiff (0.073 ± 0.075 mV, *P* = 0.003). This confirms our hypothesis and replicates the results of previous studies of stretch reflex modulation under similar task conditions.

Hypothesis 2: that inhibiting the contralateral (right) primary motor cortex would reduce the amplitude of the LLSR.

**Figure 3:**
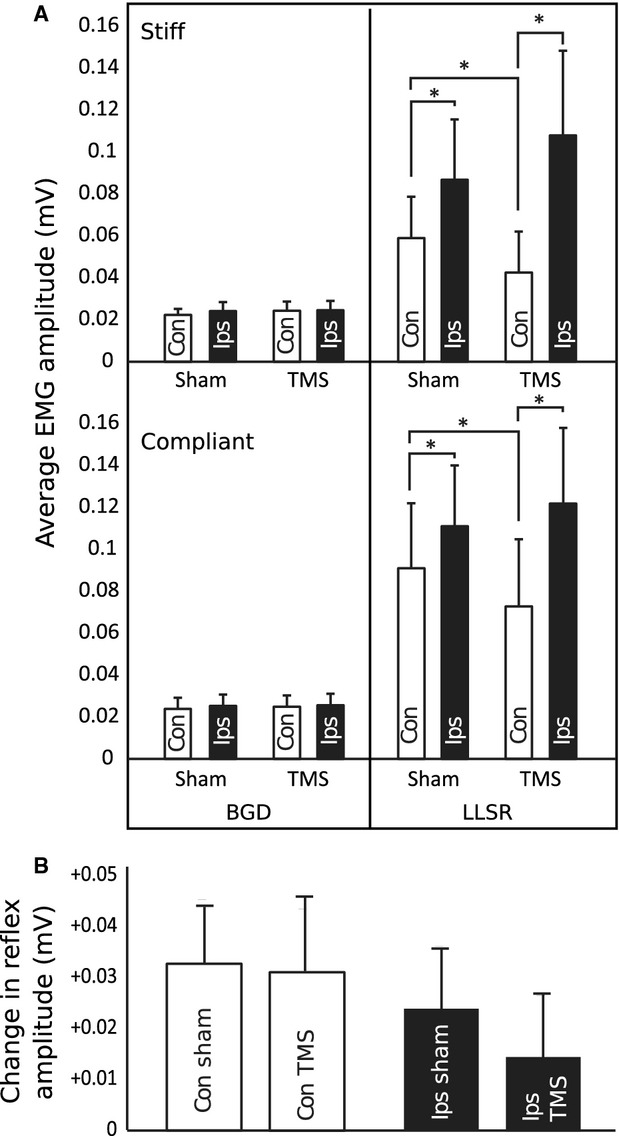
(A) The average amplitude of recorded EMG is shown during time periods representing tonic muscle activation prior to any stimuli (background [BGD]) and the LLSR. Responses following stimulation of the contralateral (right) motor cortex are designated by unfilled columns and responses to stimulation of the ipsilateral (left) motor cortex by filled columns. Tonic muscle activity was specifically matched between conditions. (B) The difference between LLSR amplitudes in the stiff and compliant environments is highlighted. This plot uses data available in (A) to more clearly compare reflex responses in the two mechanical environments. A positive change in reflex amplitude reflects larger LLSRs in the compliant compared to the stiff environment. No significant changes in LLSR modulation were observed across the four stimulation conditions. Error bars represent the standard error of the mean. Asterisks represent statistically significant changes in EMG responses (*P* < 0.05). EMG, electromyograph; LLSR, long-latency stretch reflex.

Consistent with our hypothesis, the application of supramotor threshold TMS to the primary motor cortex contralateral to the target ECR muscle 50 msec prior to wrist perturbations resulted in a period of corticospinal inhibition (Fig. 1B) and reduced the amplitude of the LLSR within the period of induced inhibition (Fig. 2A and C). Reductions in the amplitude of the LLSR were observed in both stiff (sham: 0.059 ± 0.063 mV, TMS: 0.04 ± 0.062 mV; *P* = 0.025) and compliant (sham: 0.091 ± 0.098 mV, TMS: 0.073 ± 0.010 mV; *P* = 0.036) environments (Fig. 3A and B).

Hypothesis 3: that inhibiting the ipsilateral (left) primary motor cortex would reduce the amplitude of the LLSR.

Contrary to our hypothesis, applying supramotor threshold TMS to the primary motor cortex ipsilateral to the target ECR did not reduce the amplitude of the LLSR in either mechanical environment (Fig. 2B and D). The amplitude of LLSRs induced within the period of ipsilateral motor cortex inhibition was not different to that of LLSRs induced during sham stimulation in either stiff (sham: 0.087 ± 0.091 mV, TMS: 0.108 ± 0.128 mV; *P* = 0.152) or compliant (sham: 0.111 ± 0.092 mV, TMS: 0.122 ± 0.114 mV; *P* = 0.27) environments. Interestingly, LLSR amplitude was greater when sham TMS was applied to the ipsilateral (stiff: 0.087 ± 0.091 mV, compliant: 0.111 ± 0.092 mV), compared to the contralateral (stiff: 0.059 ± 0.062 mV [*P* = 0.044], compliant: 0.091 ± 0.098 mV [*P* = 0.043]) motor cortex.

Hypothesis 4: that inhibiting the contralateral primary motor cortex would reduce modulation of the LLSR between stiff and compliant mechanical environments.

Contrary to our predictions and to evidence of the involvement of the contralateral motor cortex in LLSR gain modulation (Shemmell et al. 2009, 2010), inhibition of the contralateral hemisphere failed to reduce the change in LLSR amplitude between stiff and compliant environments (change in LLSR during sham: 0.032 ± 0.042 mV, change in LLSR during TMS: 0.030 ± 0.051 mV; *P* = 0.847; Fig. 3A and B).

Hypothesis 5: that inhibiting the ipsilateral primary motor cortex would reduce modulation of the LLSR between stiff and compliant mechanical environments.

Compared to sham, TMS-induced inhibition of the ipsilateral motor cortex did not significantly alter the extent of amplitude modulation of the LLSR between the stiff and compliant environments (change in LLSR during sham: 0.024 ± 0.033 mV, change in LLSR during TMS: 0.013 ± 0.042 mV; *P* = 0.164; Fig. 3A and B).

## Discussion

The results of this study demonstrate that, in normal participants, the contralateral but not ipsilateral motor pathway is involved in stability-dependent modulation of the LLSR in a wrist extensor muscle. The results extend previous findings suggesting that the contralateral primary motor cortex is involved in the transmission of the LLSR, although they suggest that the locus of gain regulation for this reflex response resides outside the motor cortex.

### LLSRs in wrist extensors are modulated according to the stability provided by the external environment

Previous experiments have demonstrated changes in the amplitude of the LLSR in elbow (biceps brachii) and wrist (flexor carpi radialis) flexor muscles and that are dependent on the amount of joint stability provided by the external environment (Doemges and Rack 1992; Shemmell et al. 2009). Our results show that this phenomenon also exists in muscles involved in wrist extension as, during sham TMS, the amplitude of the LLSR was greater in a compliant haptic environment than in a stiff environment. While this is unsurprising in some ways given the evidence of reflex modulation in elbow flexors and extensors, the role of flexor and extensor muscles is quite different during common reaching and grasping movements. When combined with previous evidence of reflex modulation in the upper limb, our results suggest that rapid reflex gain modulation in response to changes in the stability conferred by the environment is a capacity common to many upper limb muscles, regardless of their task-specific function. Whether this also applies to muscles primarily involved in fine control of the digits remains an open question.

### The contralateral, but not ipsilateral, primary motor cortex is involved in generating the LLSR

The present experiment found that TMS-induced suppression of activity in the right primary motor cortex reduced the size of the LLSR in the left ECR muscle in both the stiff and compliant environmental conditions. This supports previous suggestions that the motor cortex contralateral to a stretched muscle is involved in transmission of the LLSR (Kimura et al. 2006; Shemmell et al. 2009). The involvement of the motor cortex in regulating reflexes in muscles acting about the wrist has already been established (Cheney and Fetz 1984; Abbruzzese et al. 1985) and is perhaps not surprising given the large number of monosynaptic connections between the contralateral primary motor cortex and motoneurons innervating distal muscles of the upper limb (Nudo and Masterton 1990).

In contrast, suppression of activity in the left primary motor cortex had no significant effect on the amplitude of the LLSR in the left ECR. This result runs counter to our hypothesis and suggests that despite the existence of corticospinal neurons descending from the left motor cortex to motoneurons originating in the left side of the spinal cord, this pathway does not play a significant role in the regulation of the LLSR. Indeed, if suppression of the left motor cortex had any effect it was to increase LLSR amplitude, which would be consistent with evidence of reciprocal inhibitory effects of each motor cortex on the other (Ferbert et al. 1992). The extent of this reflex amplification, however, was not statistically significant and a similar increase in LLSR amplitude was observed following sham stimulation. Given the relatively high-intensity stimuli used in this experiment, it is possible that the auditory effect of TMS could activate brainstem startle reflex circuits sufficiently to release a prepared motor command. The potential for auditory stimuli to hasten prepared voluntary motor commands has been demonstrated in humans (Rothwell et al. 2002; MacKinnon et al. 2007) and as participants were asked to counteract a bias force toward wrist flexion, we know that a command for wrist extension (ECR contraction) existed prior to each stimulus. Also, in support of this idea is evidence that TMS is capable of eliciting short-latency responses in neurons of the reticular formation in monkeys (Fisher et al. 2012). It is not clear, however, whether the activation of startle reflex circuits is responsible for the difference in LLSR amplitude between TMS of the contralateral and ipsilateral hemispheres. While there is some evidence that muscular responses to startle circuit activation are lateralized (Grillon and Davis 1995), the preferential activation of muscles ipsilateral to the auditory stimulus is yet to be demonstrated. Taken together, our results and those of previous studies suggest that the primary motor cortex ipsilateral to a perturbed wrist is not involved in generation or modulation of the LLSR, although there is some suggestion that it could play a role in regulating the reflex through transcallosal inhibitory effects.

### Neither primary motor cortex regulates the gain of the LLSRs in wrist extensor muscles

While TMS-induced suppression of the right motor cortex reduced the amplitude of the LLSR, it did not reduce the stability-dependent modulation of the reflex between stiff and compliant conditions. Our hypothesis that this suppression would reduce stability-dependent modulation of the LLSR was based on previous findings demonstrating that LLSR modulation in more proximal muscles is reduced during similar TMS-induced suppression of activity in the primary motor cortex (Kimura et al. 2006; Shemmell et al. 2009). During movement, motor cortex suppression appears to eliminate modulation of the LLSR that is due to anticipated arm perturbations (Kimura et al. 2006). During postural maintenance, the same motor cortex suppression reduces LLSR modulation that occurs due to changes in environmental stability, but does not eliminate it entirely (Shemmell et al. 2009). Our current results demonstrate that stability-related LLSR modulation in a more distal muscle, the ECR, is not reduced by motor cortex suppression. When considered in the context of previous findings, our results support the idea that when the goal of a task is to maintain a consistent posture, the primary motor cortex is involved in the transmission of a transcortical stretch reflex but is not the primary locus of reflex gain regulation. The nature of motor cortex involvement may change during movement, where it appears to assume more responsibility for regulating rapid corrective actions (Fromm and Evarts 1977; Maier et al. 1993), although this is not likely achieved through reflex regulation as stretch reflexes are inhibited during the corrective phase of rapid movements (Gottlieb et al. 1983).

### Implications for motor disorder following cerebral infarction

It has recently been demonstrated that stability-dependent modulation of the LLSR is impaired in both paretic and nonparetic arms following monohemispheric stroke (Trumbower et al. 2013). As the ipsilateral motor cortex is not involved in generation or modulation of the LLSR in healthy nervous systems, it is difficult to explain why the arm ipsilateral to a stroke lesion displays impairments of reflex modulation almost as severe as the paretic arm. The bilateral deficits in reflex control evident following stroke may be due to organizational changes in the motor system that occur in response to the injury. Specifically, survivors of monohemispheric stroke demonstrate increases in the extent to which they engage the ipsilateral sensorimotor cortex during activation of their paretic arm (Netz et al. 1997; Cramer 2008). While this type of gross reorganization has been suggested to be maladaptive, it likely represents a compensatory mechanism intended to recruit neural resources from the nonlesioned hemisphere to aid in control of the paretic limb. Sharing of resources in the undamaged motor cortex may result in a reduction in the number of neurons responsible for voluntary control and reflex regulation of the nonparetic arm. While this hypothesis is speculative, it would be of interest to investigate the relative representation and function of the nonparetic arm to determine whether LLSR modulation correlates negatively with the area of ipsilateral representation.

Any implications of the current results for rehabilitative methods following stroke are necessarily highly speculative, although the lack of reflex regulation by the ipsilateral motor cortex perhaps demonstrates the importance of maximizing the use of surviving neural resources in the contralateral hemisphere. In this context, the development of experimental techniques designed to maximize the survival of neurons in the perilesional area immediately after stroke events and to encourage movement-specific reorganization within the lesioned motor cortex is both exciting and important.

## Conclusion

In summary, the present results confirm the involvement of the primary motor cortex contralateral to a target arm in stability-dependent modulation of the LLSR in healthy individuals, while denying a role for the ipsilateral motor cortex. These results imply that bilateral deficits of reflex regulation following monohemispheric stroke are not the direct result of damage to an existing bilateral reflex pathway.
